# Frequency, Awareness, and Symptoms of Chikungunya Among Patients in a Tertiary Care Hospital of Karachi: A Cross-Sectional Study

**DOI:** 10.7759/cureus.4054

**Published:** 2019-02-12

**Authors:** Zoha Mehdi, Hareem Shahbaz, Aatika Owais, Syeda Ushna Hasan, Ifrah Nasr, Ahmed Jahangir, Nashmia Zubair, Syed Abdullah Abdul Khaliq, Maria Khalid, Sana Shahbaz, Momina Qureshi, Raazia Hasan, Muhammad Fasih, Adeel Khalid, Danial Hasan, Soofia Nigar

**Affiliations:** 1 Internal Medicine, Dow University of Health Sciences (DUHS), Karachi, PAK

**Keywords:** chikungunya, frequency, viral fever, viral arthralgia, mosquito-borne infections, tertiary care hospital, karachi, pakistan

## Abstract

Background

Chikungunya fever (CHIKF) is an infectious illness spread by the bite of mosquitoes and caused by an arbovirus known as Chikungunya virus (CHIKV). This disease has become an epidemic in Karachi and various other cities of Pakistan, affecting a large population, especially those from poor, socioeconomically underdeveloped areas. It is proving to be a severe and alarming cause of debility due to its prolonged detrimental effects on the joints. A significant number of cases are reported daily in different hospitals of Karachi, with Civil Hospital being one of the major tertiary care hospitals. The aim of this study is to determine the frequency and symptoms of chikungunya as well as to assess the participants’ awareness about the spread and preventive measures of this disease.

Methods

This is a cross-sectional study that was carried out in Civil Hospital Karachi by approaching patients in the out-patient department (OPD) and the emergency department with complaints of fever and joint pain. All the data was collected via a pre-coded questionnaire during May-June 2017 by taking prior informed verbal consent and were analyzed through Statistical Package for the Social Sciences (SPSS) version 22 software.

Results

The age group most affected by this disease was the 21-30 years range, which represented almost a quarter of the cases (n=83, 32.17%). Majority of the respondents (n=214, 82.95%) had heard of the disease, mainly due to their own prior experience with it (n=100, 38.76%). Lethargy (n=219, 84.88%), difficulty in walking (n=213, 82.56%), and headache (n=209, 81.01%) were the major symptoms reported apart from fever (n=258, 100.00%) and arthralgia (n=258, 100.00%).

Conclusion

CHIKF is proving to be a great threat to people as it impairs their quality of life to a great extent. The recent outbreak of chikungunya has victimized a considerable population of Karachi. This study mainly assessed the severity of the disease and its symptoms as well as the lack of awareness among patients. Proper and effective preventive measures can further help to eradicate this disease on a large scale and prevent future epidemics.

## Introduction

Chikungunya virus (CHIKV) is a single-stranded, enveloped ribonucleic acid (RNA) arbovirus that is transmitted by the Aedes aegypti mosquito. It causes acute fever and acute and chronic articular manifestations such as the exacerbation of arthralgias, inflammatory polyarthritis, and joint stiffness [1]. Other symptoms include muscle pain, headache, nausea, fatigue, and rash. Morbidity and temporary disability occur in larger proportions due to residual joint pain. Though mortality is rare with this disease, many patients also develop neurological, cardiovascular, pulmonary, renal, ocular, and cutaneous sequelae following acute infection [2-3].

The word chikungunya comes from Makonde, a language spoken in Tanzania, which means 'walking bent over' or 'bent walker', referring to the posture patients assume with the resulting arthralgia [4]. CHIKV has spread to 22 countries including Pakistan. A few patients affected with chikungunya were also reported in Lahore during the 2011 dengue outbreak. The current outbreak is said to have started during the second week of December 2016. The total number of patients estimated by different health-care authorities in Karachi is more than 30,000. The Ministry of National Health Services, Regulation and Coordination (NHSRC), for the first time on 26 December 2016, officially reported the outbreak to the World Health Organization (WHO) [5-6].

Little work has been done to know the recent prevalence of chikungunya fever (CHIKF) in Karachi. Many cases with symptoms suggestive of CHIKF were reported in Civil Hospital, a major tertiary care hospital of Karachi. Therefore, this study was conducted to determine the prevalence of the current epidemic of CHIKF in Karachi as well as to recognize the factors responsible for the re-emergence of this virus, perhaps the disease, and to understand the factors contributing to its outbreak.

The main objectives of our study were to estimate the frequency of CHIKF during the month of May and June 2017 in Civil Hospital, Karachi, using a syndromic approach, and to check awareness of CHIKF among patients who have been in contact with the disease. We have also studied the sequelae of CHIKF and assessed common treatment-seeking behavior and control measures in the population using a cross-sectional study.

## Materials and methods

This study was carried out as a cross-sectional study in Civil Hospital, Karachi, on suspected patients who had visited the out-patient department (OPD) and emergency department (ED) with complaints of fever and joint pain and had confirmed their diagnosis, usually clinical, of chikungunya viral fever. The study was conducted using 300 patients as a sample size, which was calculated using OpenEpi software (Open Source Epidemiologic Statistics for Public Health) with a confidence limit of 5% and a confidence level of 95%. Patients who had similar symptoms but had a different diagnosis, or those lacking any of the two cardinal symptoms of the disease, that is, fever and joint pain, were excluded from the study. A non-probability sampling technique was used to enroll patients with a febrile illness consistent with joint pain and other clinical symptoms of chikungunya. Out of the 300 patients, 258 were selected for this study, giving a response rate of 85%. The importance of the study was explained at the beginning of the questionnaire-based interview and informed verbal consent was taken prior to it.

The data along with all the clinical features were collected via a pre-coded questionnaire over a period of two months (May-June 2017). The interviewers also translated the questions into the local language for the convenience of participants who were unable to comprehend English. The first half of the questionnaire inquired about the patients’ age, gender, residence, co-morbidity, and assessed their knowledge regarding CHIKV; specifically, its mode of spreading, symptomatology, complications, and preventive measures. The participants were also briefly counselled about the above-mentioned specifics, especially the preventive modalities they can employ. The second part of the questionnaire contained questions that had to be answered with a 'yes' or 'no', and mainly focused on the clinical signs and symptoms observed in suspected patients. The duration of each symptom was also noted alongside with it. All the findings were recorded, and data analysis was done later using Statistical Package for the Social Sciences (SPSS) software version 22 (IBM Corporation, USA, Armonk, NY).

## Results

Demographics

**Figure 1:**
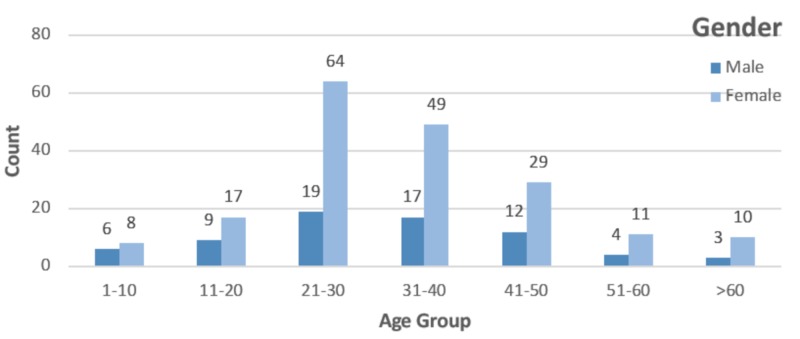
Age-wise and gender-wise distribution

The mean age of our participants was 33.62 years (range = 1-60 or above). Out of the total of 258 eligible participants, only 2.33% (n=6) presented to the emergency department, while the remaining 97.67% (n=252) presented to the OPD. The age group most affected was the 21-30 years age range, which represented almost a quarter of the cases (n=83, 32.17%). The remaining demographic data has been provided in Table 1. An age group and sex-wise distribution of cases has been illustrated in Figure 1.

**Table 1:** Summary of demographic characteristics

Demographics	Frequency (n)	Percent (%)
GENDER		
Male	70	27.13
Female	188	72.87
MARITAL STATUS		
Single	62	24.03
Married	186	72.09
Divorced	2	0.78
Widowed	8	3.10
EDUCATION		
Primary	19	7.36
Secondary	43	16.67
Higher education	5	1.94
None	191	74.03
ETHNICITY		
Urban	240	93.02
Rural	18	6.98
CHRONIC DISEASE STATUS		
Diabetes	14	5.43
Hypertension	29	11.24
Heart disease	4	1.55
Lung disease	4	1.55
Others	12	4.65
More than one of the above	20	7.75
None	175	67.83
HEALTH SEEKING BEHAVIOR		
Out-patient department	252	97.67
Emergency department	6	2.33

Knowledge of chikungunya

**Figure 2:**
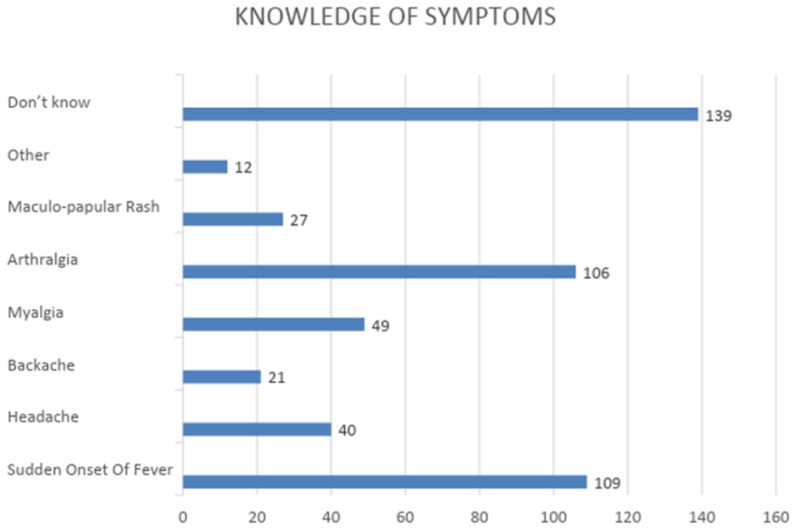
Knowledge of symptoms

**Figure 3:**
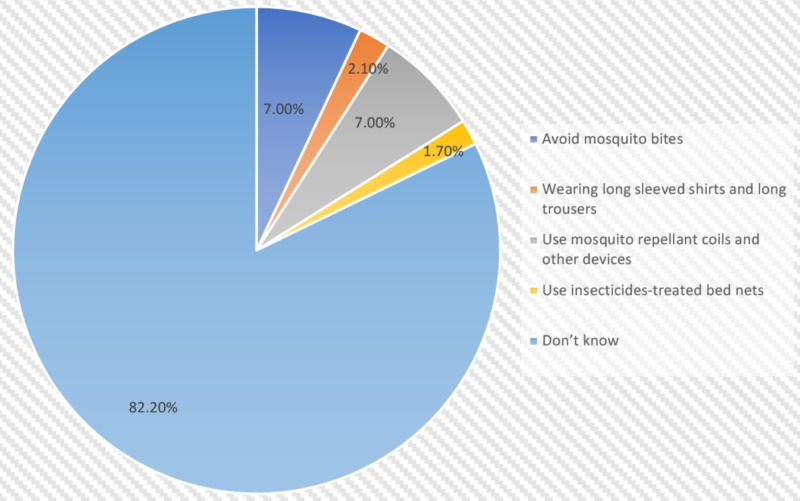
Knowledge regarding methods of prevention

Majority of the respondents (n=214, 82.95%) had heard about chikungunya, the major source being their own prior experience with the disease (n=100, 38.76%). Others were aware because of a family member (n=50, 19.38%), or someone they knew had contracted the disease (n=28, 10.85%), while the remaining (n=36, 13.95%) had gained awareness through some other means. Only one-sixth (n=46, 17.83%) knew the correct mode of transmission of the disease. Figure 2 shows the knowledge of the participants regarding the various symptoms of the disease in which fever (n=109, 42.25%) and arthralgia (n=106, 41.09%) were the major responses. Almost all the participants (n=254, 98.45%) were unaware of the long-term complications of chikungunya, the remaining (n=4, 1.55%) responded with gastrointestinal complications (n=1, 0.39%), meningoencephalitis (n=2, 0.78%), and others (n=1, 0.39%). Figure 3 is an illustration of the various methods of prevention that participants believe are effective against the disease. No one chose the option of setting the air conditioning to a low temperature at night as a method of prevention (n=0, 0.00%).

Symptomatic inquiry

Table 2 shows the symptoms of the patients. Fever and arthralgia were the most consistent symptoms present in all the respondents (n=258, 100.00%), with a majority (n=189, 73.26%) of the participants suffering from fever for a duration of less than seven days, almost one-fifth (n=47, 18.22%) suffering for a month, while the remaining suffered for two (n=18, 6.98%) or more than two months (n=4, 1.55%). Lethargy (n=219, 84.88%), difficulty in walking (n=213, 82.56%), and headache (n=209, 81.01%) were the major symptoms reported apart from fever and arthralgia, with weight gain (n=24, 9.30%) being the least common symptom reported. The mean duration of the symptoms is shown in Table 3.

**Table 2:** Summary of symptomatic inquiry

	Responses	Frequency (n)	Percent of cases (%)
SYMPTOMATIC INQUIRY	Fever	258	100.00
	Headache	209	81.01
	Arthralgia	258	100.00
	Abdominal pain	81	31.40
	Nausea	122	47.29
	Vomiting	87	33.72
	Oral ulcers	69	26.74
	Weight gain	24	9.30
	Weight loss	48	18.60
	Lethargy	219	84.88
	Anorexia	178	68.99
	Diarrhea	41	15.89
	Giddiness	150	58.14
	Morning stiffness	213	82.56
	Joint stiffness	195	75.58
	Sleep disturbances	157	60.85
	Difficulty in walking	213	82.56
	Myalgia	158	61.24
	Retro-orbital pain	107	41.47
	Eye congestion	100	38.76
	Chills	183	70.93
	Cough	49	18.99
	Runny nose	36	13.95
	Edema	140	54.26
	Rashes	91	35.27

**Table 3:** Mean and standard deviation of the duration of symptoms

Symptom duration	Frequency (n)	Mean (days)	Standard deviation
Fever duration	258	12.00	18.61
Headache duration	209	15.36	22.48
Abdominal pain duration	82	15.94	22.38
Nausea duration	122	13.36	17.78
Vomiting duration	87	7.35	13.78
Oral ulcers duration	69	12.57	17.93
Lethargy duration	220	22.79	45.90
Anorexia duration	178	18.33	22.57
Diarrhea duration	42	16.33	24.37
Giddiness duration	149	17.47	21.89
Morning stiffness duration	213	23.01	26.46
Joint stiffness duration	195	25.26	34.92
Sleep disturbance duration	157	20.40	25.74
Difficulty in walking duration	215	21.55	26.16
Myalgia duration	157	21.63	25.88
Retro-orbital pain duration	107	11.27	16.24
Eye congestion duration	100	11.33	16.36
Chills duration	184	10.34	15.77
Cough duration	50	19.24	25.25
Runny nose duration	36	12.56	20.96
Arthralgia duration	258	23.87	44.80
Edema duration	140	20.14	24.77
Rashes duration	92	15.76	22.74

**Figure 4:**
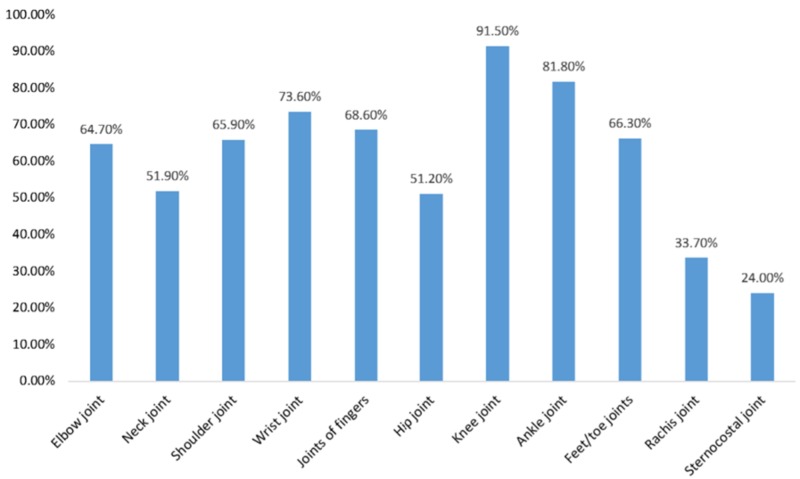
Joints affected by arthralgia

**Figure 5:**
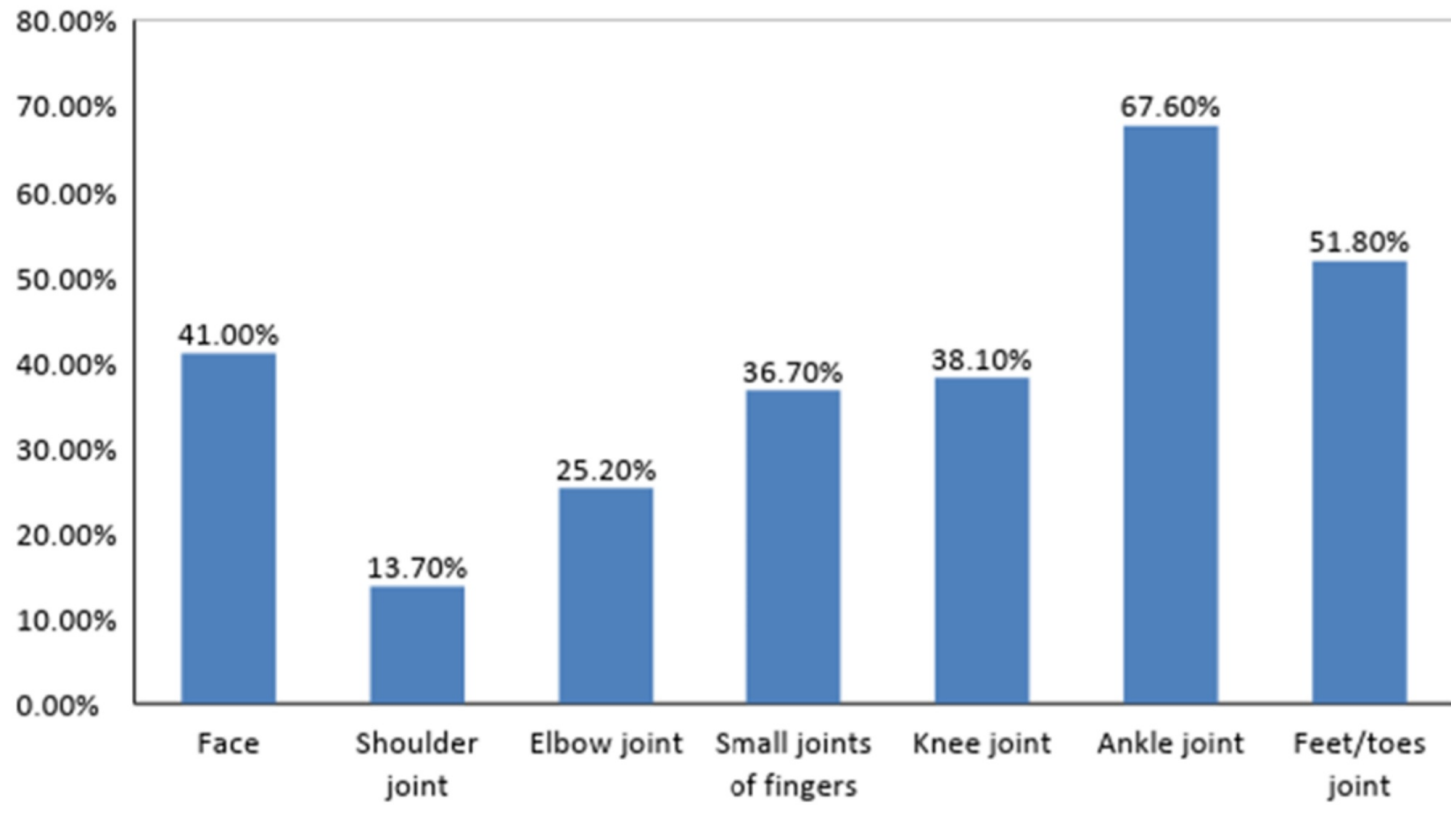
Joints affected by edema

**Figure 6:**
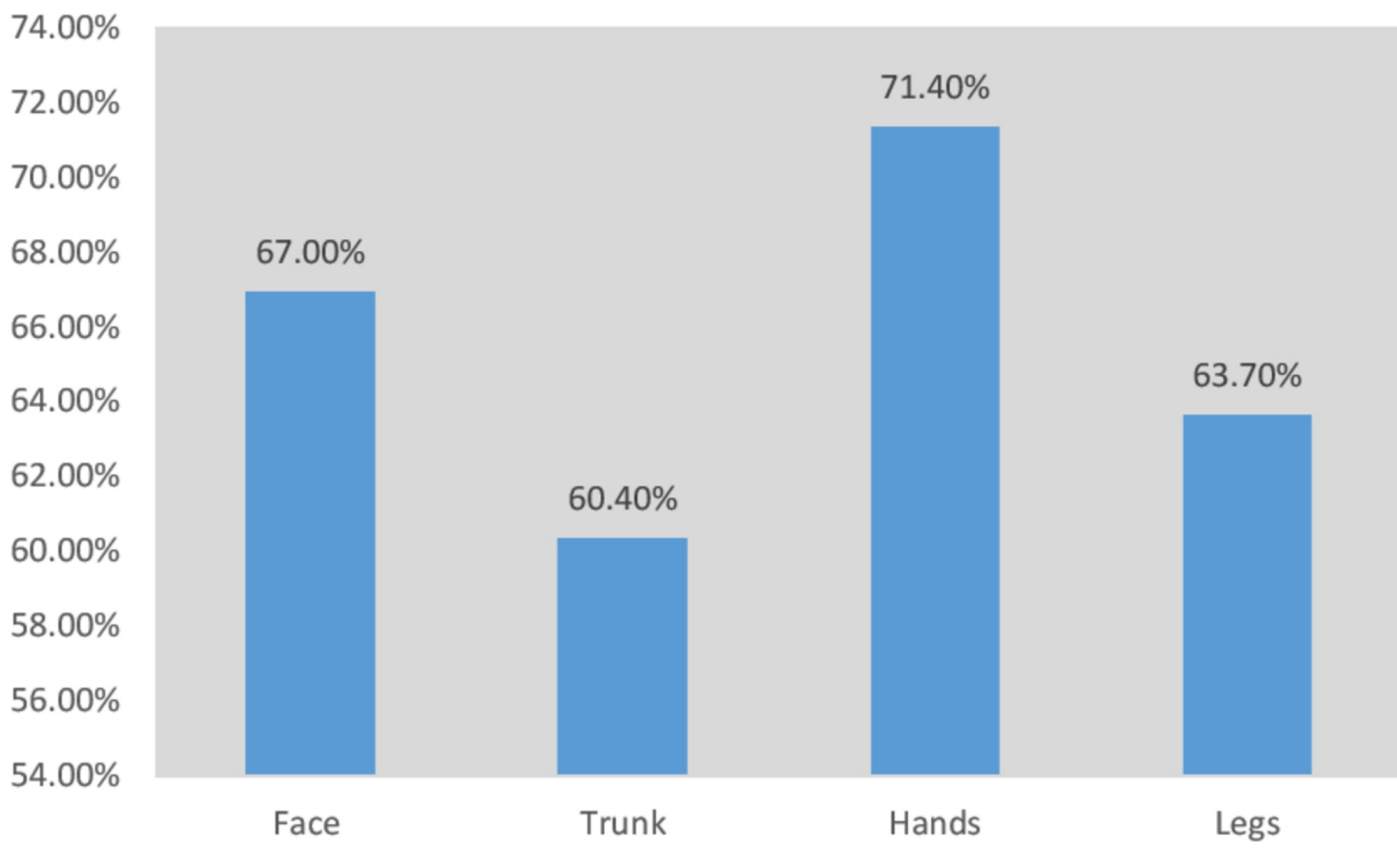
Localization of rash

More than half of the participants (n=179, 69.38%) graded the intensity of their joint pain as severe, while almost a quarter of the participants (n=64, 24.81%) graded it as moderate and the remaining (n=15, 5.81%) as mild. The duration of arthralgia noted is written in Table 4. Figure 4 and Figure 5 shows the frequencies at which joints were affected by arthralgia and edema, respectively. Figure 6 shows the localization of rash.

**Table 4:** Duration of arthralgia

Duration	Frequency (n)	Percent (%)
Less than 7 days	134	51.94
8-30 days	76	29.46
31-60 days	33	12.79
61-90 days	9	3.49
>90 days	6	2.33
Total	258	100.00

## Discussion

Out of the 258 participants who were selected for the study, a majority of the participants were female (n=188, 72.87%), which is contrary to the findings of three relevant studies [7-9]. All age groups were affected, but a majority of the patients belonged to the age group 21-40 years (n=83 patients from the age group 21-30 years; and n=66 patients from the age group 31-40 years) similar to the results of a study done in Andhra Pradesh, South India (age group 21-50 years) [8]. A majority of the patients presented in the OPD (n= 252, 97.67%), similar to a study done in Maharashtra, India [10]; this indicates that their symptoms were not severe or pertinent enough to warrant immediate health care.

Affected patients that presented in the OPD mainly belonged to urban but medium-to-low socioeconomic status areas. The contributing factors are most likely related to their polluted environment, which is common among most of the areas reported to be harboring an epidemic of chikungunya. Most of these areas have unsatisfactory drainage systems, owing to which the overflowing gutters and stagnant water not only add to the pollution of the environment, but they also invite mosquitoes, the vectors for CHIKV, to dwell and reproduce. Open sewers and stagnant water bodies, which make excellent breeding grounds for mosquitoes, can be found all throughout the city. There is an urgent need to rid the city of these favorable habitats and to raise awareness via campaigns [5]. Another contributing factor can be the relatively lower level of literacy and decreased drive for education in the resident population, which can subsequently result in indifference to the maintenance of hygiene and cleanliness in the environment as well as a lack of awareness about the chikungunya outbreak in other parts of Karachi.

A majority of the participants (n=214, 82.95%) reported having an awareness of the disease, which is similar to a study undertaken in a rural population of Kasegaon village in Maharashtra, India [7]. The source of information was mainly cited as the participant's own prior experience with the disease (n=100, 38.76%) and only 17.80% (n=46) participants were aware that mosquitoes were vectors for chikungunya which was in contrast to a study done in a tertiary care institute in Telangana state, India, which showed that 88.89% of participants were aware that mosquitoes were vectors [11]. Around 46.21% of our study population had some knowledge of the signs and symptoms of chikungunya, which differs from another study done in Gujarat, India, according to which only 22% of the population knew the correct signs and symptoms associated with this disease [12]. A lack of awareness regarding methods of prevention was found in 82.20% of our study population, as compared to another study that showed that only 20.55% of the population was unaware of methods of prevention [13].

The most commonly reported symptoms were fever and arthralgia, which were reported by all participants (n=258, 100.00%). Similar findings were reported by another study, according to which the sudden onset of fever of short duration (100% cases), followed by severe and disabling arthritis, which involves the knee, ankle, wrist, and small joints of hands and feet (98% cases), were the most significant clinical manifestations [14]. The fever was reported to have lasted for a mean duration of 12 days (range = 1-120 days), which contrasts two other studies reporting the duration of fever as three to five days and four days, respectively [15-16]. Associated with fever, 81.01% (n=209) complained of a headache and 61.24% (n=158) complained of myalgias, which were in contrast to another study that reported the prevalence of the above symptoms as 97.5% and 99.7%, respectively [17]. According to a study, lymphadenopathy, pruritus, and digestive abnormalities may also be reported but are less common [18]. The joint affected by arthralgia most commonly was the knee joint (91.50%), which is affirmed by the results of another study, but is in contrast to the results of a study that reported the involvement of predominantly distal joints [7,15]. The duration of arthralgia was also found to be less than seven days in a majority of the cases (n=134, 51.94%), which differs from a study that reported the manifestation of arthralgia as resolving within one to four months [19]. A similar study reported that fever and arthralgia can only be relieved by rest, hydration, proper diet, exercise, and analgesics such as ibuprofen, naproxen, acetaminophen, or paracetamol, provided that the person has no contra-indications to these medications [20].

Half of the population (n=140, 54.26%) reported edema, which most commonly affected the ankle joints (67.60%), and similar findings were reported in a study done in Reunion Island, in which 50% of the study population reported edema mainly involving the ankle joint (70%) [21]. Many of our patients who reported edema as a symptom had more than one affected region. Around 35.27% of the population also reported the occurrence of rashes, mainly affecting the hands (71.40%), which differs from a relevant study showing generalized lesions, with localized erythema of the nose in 15% of the patients [22]. Again, most of the patients who reported rashes as a symptom had more than one region affected. On the contrary, a recent research study done on chikungunya in children revealed rashes as the most consistent symptom (66%) [23]. According to similar studies, skin rashes were present in about 40%-50% of cases, which usually appear between the second and fifth day of onset of fever and are mostly maculo-papular and pruritic, with maculo-papular rashes being most common on the face and trunk [15,24].

Limitations

Our study is limited to the patient population of Civil Hospital, Karachi. In addition, due to the poor socioeconomic status of the patients and excessive costs of the polymerase chain reaction (PCR) immunoglobulin M (IgM) and immunoglobulin G (IgG) tests for chikungunya, a confirmed diagnosis could not be acquired. The patients were diagnosed by general physicians based only on blood investigations and symptomatology. Furthermore, the specific PCR diagnostic test for CHIKV is now available free of cost in Civil Hospital Karachi effective from September 2017, and future studies on frequency can be done without the above limitation.

Future recommendations

Chikungunya causes temporary disability in patients by impairing their normal routine and professional lives. Therefore, it is highly recommended to develop a vaccine against this disease to control its recent outbreak throughout the world. The sanitary conditions of neglected areas in both urban and rural populations should be improved. Awareness programmes regarding the spread and prevention of this disease should be conducted. The government must provide for the eradication of mosquito-breeding sites and the implementation of effective vector-control methods; it should also minimize the cost of the diagnostic test for chikungunya in all the major tertiary care centres to make it more affordable for the population.

## Conclusions

Chikungunya is an infectious viral illness that impairs the personal and professional lives of the people affected. Its clinical features and complications are widespread, requiring timely and effective management. The recent outbreak of chikungunya in Pakistan, and especially in Karachi, has affected the population to a dangerous extent. Lack of awareness is the main barrier to disease prevention. Through proper awareness programmes and the eradication of vector breeding sites, we can prevent future epidemics of chikungunya.
